# Antiphospholipid Syndrome-Induced Leriche Syndrome in a Man with Lower Limbs Sensory and Motor Defect

**DOI:** 10.3390/jcdd8090104

**Published:** 2021-08-29

**Authors:** Jeng-Luen Hong, Yueh-Tseng Hou, Po-Chen Lin, Yu-Long Chen, Da-Sen Chien, Giou-Teng Yiang, Meng-Yu Wu

**Affiliations:** 1 Department of Emergency Medicine, Taipei Tzu Chi Hospital, Buddhist Tzu Chi Medical Foundation, New Taipei 231, Taiwan; 97311126@gms.tcu.edu.tw (J.-L.H.); brianann75@gmail.com (Y.-T.H.); taipeitzuchier@gmail.com (P.-C.L.); yulong0129@gmail.com (Y.-L.C.); sam.jan1978@msa.hinet.net (D.-S.C.); gtyiang@gmail.com (G.-T.Y.); 2 Department of Emergency Medicine, School of Medicine, Tzu Chi University, Hualien 970, Taiwan

**Keywords:** antiphospholipid syndrome, acute aorto-iliac occlusive disease, Leriche syndrome, acute thrombosis

## Abstract

Antiphospholipid syndrome (APS) is an autoimmune disorder with characteristics of arterial and/or venous thrombosis due to hypercoagulation status. Although deep vein thrombosis is common, the involvement of arterial thrombosis is more dangerous and poses a high risk of complications. Acute aorto-iliac occlusive disease (AIOD, known as Leriche syndrome) is severe arterial thrombosis that is associated with high morbidity and mortality rates. Severe acute occlusion may cause spinal cord ischemia, leading to neurological defects, such as acute onset of paraplegia. Co-occurrence of acute aorto-iliac occlusive disease and antiphospholipid syndrome is rare and may present with atypical symptoms mimicking other diseases, including chronic ulcers, musculoskeletal events, and pulmonary diseases. In patients with weak femoral pulses and recurrent thrombotic events, co-occurrence of APS and AIOD should be taken into consideration. Here, we describe a rare case of co-occurrence of APS and AIOD presenting with acute lower leg weakness and numbness. Timely thrombectomies and bilateral common iliac artery stentings rescued distal blood flow. We highlight the clinical features and early diagnosis of co-occurrence of APS and AIOD in order to prevent catastrophic complications. The detailed mechanism and pathogenesis of antiphospholipid syndrome-induced acute aorto-iliac occlusive disease are also discussed.

## 1. Introduction

Acute aorto-iliac occlusive disease (AIOD), known as Leriche syndrome, is a rare but dangerous vascular emergency associated with high morbidity and mortality rates. The clinical triad of AIOD was reported as buttock claudication, erectile dysfunction, and absent femoral pulses [1]. Other symptoms include pain, pallor, pulselessness, paralysis, and paresthesias [2]. Etiology may be caused by coagulopathy and hypercoagulation status. Severe acute occlusion may cause spinal cord ischemia, leading to neurological defects, such as acute onset of paraplegia. Clinically, it is difficult to differentiate between vascular or neurogenic origin of acute neurological defect and paraplegia. Although aortic occlusion is very rare, there is a need to keep it in mind as a potential possibility in order to avoid misdiagnosis of a major vascular event and prevent adverse outcomes associated with delayed diagnosis. Early diagnosis is very important for physicians to ensure timely surgical intervention in antiphospholipid syndrome-induced AIOD. 

Antiphospholipid syndrome (APS) is an autoimmune disorder with characteristics of arterial and/or venous thrombosis. The diagnosis of APS is based on clinical features of thrombosis events and the presence of antiphospholipid antibodies, including lupus anticoagulant, anticardiolipin antibodies, or anti-β2 glycoprotein 1 antibodies [3]. The “two-hit” model has been promoted for pathophysiology of antiphospholipid antibody-associated thrombosis. The presence of antiphospholipid antibodies is the first hit for thrombotic complications due to an increase in thrombotic response via the priming of immune cells, platelets, and endothelial cells. The second hit is endothelial injury as a key to the activation of complement and coagulation, which strongly accelerates the formation of a thrombus [4]. The development of vascular thrombosis may cause pregnancy morbidity, stroke, myocardial infarction, and deep vein thrombosis. In R. Cervera et al.’s observational study [5], it was observed that APS-associated thrombotic complications could occur in every blood vessel, although deep vein thrombosis is more common. However, APS associated with AIOD is a very rare condition, and there is a scarcity of literature focusing on this disease. Therefore, in this paper, we describe a rare case of antiphospholipid syndrome-induced AIOD presenting with acute lower leg weakness and numbness. The clinical features and pathogenesis of antiphospholipid syndrome-induced acute aorto-iliac occlusive disease are discussed. 

## 2. Case Presentation

### 2.1. Interview

A 36-year-old male patient presented with acute onset dizziness during work. He had poorly controlled type II diabetes mellitus for ten years and deep vein thrombosis in his right thigh two years earlier. The associated symptoms included left lower limb weakness and numbness. There was no recent traumatic injury. He also denied having a headache, fever, dyspnea, nausea, vomit, chest pain, or abdominal pain. 

### 2.2. Physical Examination

On physical examination, his temperature was 36.2 °C, blood pressure was 155/78 mmHg, heart rate was 99 beats/min, and glucose testing was 288 mg/dL. The Glasgow Coma Score (GCS) demonstrated that he was aware and alert (E_4_V_5_M_6_). His breath sound was clear, and his abdomen was soft without local tenderness. A neurologic examination showed a sensory and motor defect at bilateral lower limbs. Bilateral lower leg muscle power symmetrically decreased in proximal and distal limbs. There was no cranial nerve defect. 

### 2.3. Laboratory Analysis

Initial laboratory evaluation revealed no significant infection pattern, including no increased white cell count, C-reactive protein, or neutrophil. Coagulation analysis showed no coagulopathy, and fibrin degradation product D-dimer (FDP D-dimer) test was within normal range. Biochemical analysis revealed no hepatitis, acute renal injury, or electrolyte imbalance (Table 1). The detail autoimmune antibody profile revealed positive results of APS, including lupus anticoagulant, anti-cardiolipin, and anti-β2 glycoprotein 1 antibodies (Table 2).

### 2.4. Imaging Tests 

**Figure 1:**
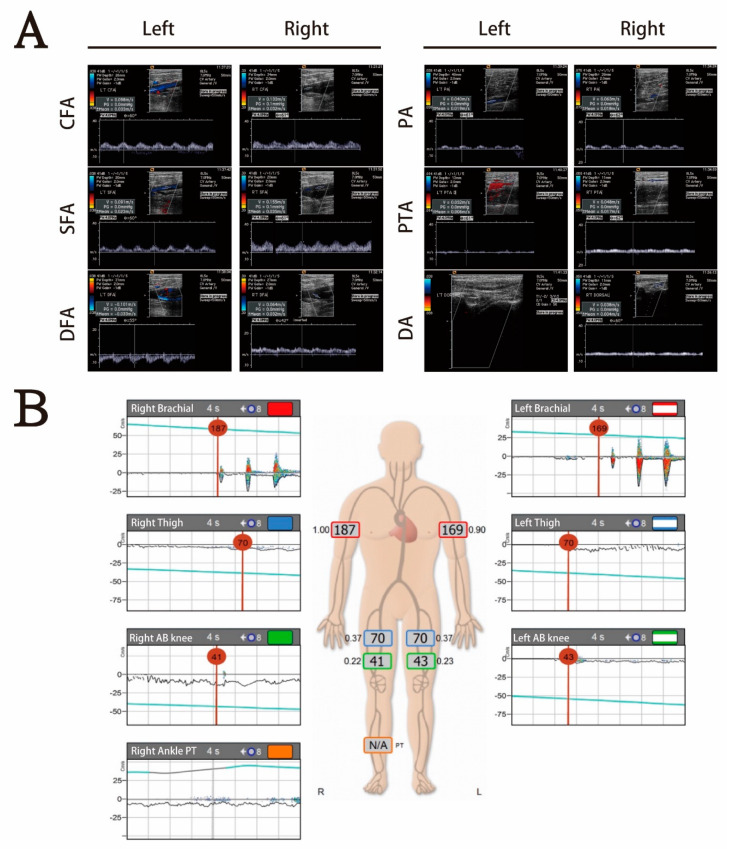
(**A**) Doppler of bilateral extremities revealed no atherosclerosis formation at bilateral femoral artery from common femoral artery to distal popliteal artery, but flow was decreased. There was no blood flow at left dorsal pedis. An upstream lesion was suspected. (**B**) The abnormal ankle-brachial index test revealed poor blood flow below the bilateral common femoral artery and suggested acute ischemia.

**Figure 2:**
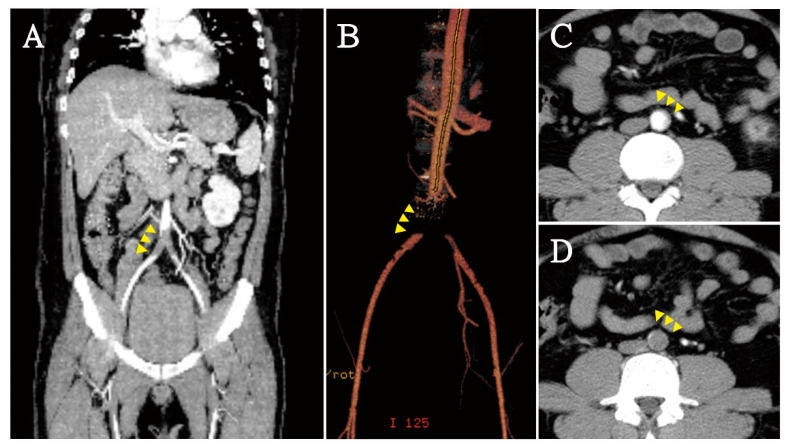
In computed tomography angiography (CTA), saddle embolism (yellow arrowhead) of distal aorta and bilateral common iliac arteries was found (**A**,**B**) in coronal plane; (**C**) partial occlusion at infrarenal aorta; (**D**) total occlusion in distal aorta.

Brain computed tomography revealed no intracranial hemorrhage, dense MCA sign, hypodense lesion, or occupied lesion. Doppler of bilateral extremities was arranged to access peripheral arterial occlusive disease (PAOD) and revealed decreased flow velocity from bilateral common femoral artery to distal popliteal artery. There was no atherosclerosis formation at bilateral femoral and popliteal artery. An upstream lesion was suggested (Figure 1). Lower extremity computed tomography angiography (CTA) showed occlusion of distal aorta and bilateral proximal common iliac arteries (Figure 2). 

### 2.5. Diagnosis and Treatment 

This patient was diagnosed with acute aorto-iliac occlusive disease (AIOD), known as Leriche syndrome, which may be caused by antiphospholipid syndrome. Recurrent thromboembolic events at young age lead us to highly suspect genetic or autoimmune disease. Antiphospholipid syndrome was diagnosed based on positive result of anti-cardiolipin IgG/IgM and anti-β2 glycoprotein 1. Emergency bilateral transfemoral Fogarty catheter thrombectomy and bilateral common iliac artery stentings were done to rescue distal blood flow. After surgical intervention, follow-up CTA revealed proper vascular graft at abdominal aortic bifurcation and bilateral common iliac arterial regions with patency of the graft. Treatment with aspirin 100 mg per day, clopidogrel 75 mg per day, and warfarin 2.5 mg per day was prescribed in order to help prevent further thrombotic events. The patient was discharged and received outpatient department follow-up. After one year of follow-up, he presented with dyspnea and acute heart failure at our emergency department. Echocardiogram showed poor heart function (Left ventricular ejection fraction, LVEF: 23.2%) and abnormal wall motion. Cardiac catheterization exam showed akinesia of anterior wall and apex at left ventricle without coronary artery stenosis. The patient died due to multiple organ failure, even under percutaneous cardiopulmonary support (PCPS). 

## 3. Discussion

Although antiphospholipid syndrome has a broad spectrum of thrombotic and non-thrombotic features, thrombotic events are the most common symptom in the APS population. Antiphospholipid antibodies have been shown to induce platelet activation and increase adhesion and aggregation, leading to high coagulation status in APS patients. Higher serum levels of other procoagulants factors, such as von Willebrand factor, have also been shown to promote thrombosis formation [6]. In Yair Levy et al.’s study [7], 88 hospitalized patients with APS were included and classified into those with or without a history of thrombosis. The results showed that increased platelet adhesion and aggregation were found in the APS patients with history of thrombosis. At least two mechanisms of APS-induced hypercoagulation have been reported, including antiphospholipid antibodies inducing platelet activation and high platelet deposition by von Willebrand factor causing shear stress [6]. Patients with APS may have spontaneous venous or arterial thromboembolism, which may more commonly involve a venous site than a pure arterial site. Legs represent the major site of deep vein thrombosis. Arterial occlusion is less frequently reported but more dangerous, including occlusion in coronary, visceral, and peripheral arteries [8]. In severe arterial occlusion-associated APS, early revascularization is the standard therapy to prevent catastrophic complications. Cardiac involvement of APS may include the potential involvement of valve lesions, myocardial infarction, cardiac thrombi, pulmonary hypertension, and cardiomyopathy. In catastrophic APS, up to 50% of patients had cardiac involvement, especially in the form of myocardial infarction and valvular heart disease [9]. Cardiomyopathy may develop from progressive valvulopathy and remodeling from ischemic heart disease. The mechanism of APS-associated cardiomyopathy is a result of APS antibodies against endothelium integrity. Thrombotic microangiopathy occurs via cytokine storm and triggers an immune response by complement activation. The release of proinflammatory and prothrombotic cytokines injures endothelial cell membranes, causing myocardial infarction, cardiac thrombi, and valvular damage. A vicious circle of APS antibodies and persistent inflammation may lead to cardiac remodeling and progressed to cardiomyopathy. 

Co-occurrence of acute aorto-iliac occlusive disease (AIOD, known as Leriche syndrome) and antiphospholipid syndrome is rare but more dangerous than venous thrombosis. Leriche syndrome was originally described as the syndrome of thrombotic obliteration of the aortic bifurcation and is more commonly observed in men in the third to sixth decades of life [10,11]. The typical presentation occurs in male patients with the clinical triad of intermittent claudication, impotency, and absent femoral pulses [12]. However, co-occurrence of Leriche syndrome and antiphospholipid syndrome may present with vary atypical symptoms, such as chronic ulcers, musculoskeletal events, pulmonary diseases, optic neuropathy, and adrenal insufficiency. In Tai-Chin Hsieh et al.’s report [13], a man with refractory ulcers of the lower limbs with bilateral lower legs muscle atrophy was reported. In Keller et al.’s report [14], co-occurrence of Leriche syndrome and dilated cardiomyopathy was successfully treated by implanted defibrillator and surgical reconstruction. In another similar report conducted by Toffon et al. [15], a patient presented with lower limb ischemia due to AIOD related to APS. After aorto-bisiliac bypass surgical therapy, she was successfully treated with anticoagulant therapy (warfarin). Presently, early revascularization is the standard therapy of Leriche syndrome with APS. After reperfusion, controlled hypercoagulation status is important in order to help prevent recurrent thromboembolic events. Secondary thromboprophylaxis is recommended, including antiplatelet and/or anticoagulant therapy. 

## 4. Conclusions

Co-occurrence of acute aorto-iliac occlusive disease and antiphospholipid syndrome may present with atypical symptoms and mimic other diseases. In patients with weak femoral pulses and motor dysfunction, acute aorto-iliac occlusive disease should be taken into consideration. In young populations and in those with recurrent thrombotic events, the etiology of hypercoagulation needs to be investigated, especially APS. Timely intervention may prevent catastrophic complications.

## Figures and Tables

**Table 1:** The basic laboratory analysis in this patient.

Variables	Patient Data		Reference Value
White cell count	7.8	--	3.5–11 (×10^9^/L)
Neutrophil	72.9	--	40–75%
Lymphocyte	20.0	--	40–45%
Monocyte	4.5	--	2–10%
Eosinophil	2.2	--	1–6%
Hemoglobin	15.0	--	12–16 g/dL
Platelet counts	102	↓	150,000–400,000/μL
PT	9.5	--	8.0–12.0 s
APTT	29.4	--	23.9–35.5 s
INR	0.93	--	
FDP D-dimer	485.04	--	0–500 ng/mL
Blood urine nitrogen	16	--	7–18 mg/dL
Creatinine	0.9	--	0.55–1.02 mg/dL
Sodium	132	↓	136–145 mmol/L
Potassium	3.5	--	3.5–5.1 mmol/L
Glucose	232	↑	70–100 mg/dL
Alanine aminotransferase	61	--	16–63 U/L
High-sensitive troponin I	22.3	↑	0–19 ng/L
Lactate	0.4	--	0.4–2.0 mmol/L
C-reactive protein	8.32	↑	0–0.33 mg/dL
HbA1C	7.5	↑	4.0–6.0%

HbA1C: glycated hemoglobin; PT: prothrombin time; APTT: activated partial thromboplastin time; INR: international normalized ratio; FDP D-dimer: fibrin degradation product D-dimer; --: no upper or below low limit of reference range. **↑**: higher than upper limit of reference range; **↓**: below the low limit of reference range.

**Table 2:** The laboratory evaluation of autoimmune antibody analysis in this patient.

Variables	Patient Data		Reference Value
Anti-cardiolipin IgG	140	**↑**	0–10 GPL U/mL
Anti-cardiolipin IgM	17	**↑**	0–10 MPL U/mL
Anti-β2 glycoprotein 1	102	**↑**	0–10 U/mL
ANA	Negative	--	1:40–1:40
Anti SS-A	0.4	--	0–10 U/mL
Anti SS-B	0.4	--	0–10 U/mL
Anti RNP	1.4	--	0–10 U/mL
Anti Sm	1.1	--	0–10 U/mL
Anti nDNA	1.2	--	0–15 IU/mL
C 4	24.2	--	10–40 mg/dL
C 3	128	--	90–180 mg/dL
Lupus AC		--	
LA 1 (Screening)	90.6	**↑**	31–44
LA 2 (Confirmation)	39.2	**↑**	30–38
LA 1/LA 2 ratio	1.9	**↑**	0.8–1.2

ANA: antinuclear antibody; Anti SS-A (Ro): anti-Sjögren’s-syndrome-related antigen A antibody; Anti-SSB (La): anti-Sjögren’s-syndrome-related antigen B antibody; Anti RNP: anti-ribonucleoprotein antibody; Anti Sm: anti-Smith antibody, Anti nDNA: anti-double stranded DNA antibodies; Lupus AC: lupus anticoagulant; --: no upper or below low limit of reference range. **↑**: higher than upper limit of reference range; **↓**: below the low limit of reference range.

## Data Availability

Not applicable.
